# Ejaculate testosterone levels affect maternal investment in red junglefowl (*Gallus gallus gallus*)

**DOI:** 10.1038/s41598-019-48563-w

**Published:** 2019-08-20

**Authors:** Asmoro Lelono, Bernd Riedstra, Ton Groothuis

**Affiliations:** 1 Behavioural Biology, Groningen Institute for Evolutionary Life Sciences, University of Groningen, Nijenborgh 7, 9747 AG Groningen, The Netherlands; 2 Department of Biology, Faculty of Mathematics and Natural Sciences, University of Jember, 68121 Jember, East Java Indonesia

**Keywords:** Behavioural ecology, Sexual selection

## Abstract

Substantial concentrations of testosterone are not only present in a male’s circulation, but also in its ejaculate. Surprisingly, the regulation of ejaculate T and its effects on females and their offspring, potentially a cryptic paternal trait, are not known. We found lower circulating and higher ejaculate T concentrations in subordinate red junglefowl (*Gallus gallus gallus*) males compared to dominant males, suggestive of an adaptive trade-off in T allocation to circulation and their ejaculate. Subsequently, we artificially inseminated females with either testosterone enriched (TE) or control ejaculates (CE) in a cross-over design. TE females produced heavier eggs than CE females. Offspring growth and tonic immobility were affected in a sex-specific way by TE. TE sons were heavier with shorter TI duration than CE sons, and TE daughters were lighter than CE daughters but daughters did not differ in TI score. However, the chicks competitiveness was not influenced by the TE nor CE. This indicates a previously unknown function of ejaculate testosterone as well as a new form of interaction between a cryptic paternal trait and a maternal effect that may be widespread in the animal kingdom.

## Introduction

In a wide variety of plant and animal taxa, parents contribute not only genetic material but also non-genetic material to their offspring. Such parental effects, in which the phenotype of the parents affect the phenotype of the offspring, can have important short and long term effects on offspring fitness and influence the rate and direction of evolution^1–3^. Prenatal parental effects are of special interest as the embryo is especially sensitive to environmental factors and such effects can have long term consequences for the offspring. Such prenatal effects are mostly studied in terms of maternal effects because the role of the mother during early development of the offspring is often larger than that of the father. However, it is well known that fathers also contribute more than their genes to the offspring, even in species where only the female contributes to parental care. One pathway through which fathers may affect offspring is via the composition of the ejaculate. Ejaculates containing amongst other compounds, proteins, enzyme, antibody, etc. These include many proteins that have been shown to influence female reproduction, especially but not exclusively, in insects (reviewed in^4^). In addition, the ejaculate also contains steroid hormones that may affect female reproduction, as shown in the malaria mosquito (*Anopheles gambiae*)^5^. Ejaculate of the vertebrates may also contain testosterone (T). The concentration of testosterone can vary among individual males, while in vertebrates both sperm and the female reproductive tract, as well as the early embryo, contain steroid receptors (reviewed in^6–8^). However, neither the regulation nor the function of these steroid hormones in ejaculates is clear.

As far as the regulation of ejaculate hormone concentrations is concerned, the most parsimonious hypothesis is that these reflect those in the male’s circulation. Alternatively, hormone concentrations in the male’s circulation and ejaculates can be regulated independently. Such an independent regulation can be beneficial in case the differential effects of the hormone in the male’s own circulation and in the ejaculates would provide the male with a tool to adjust these effects to different contexts. For example, subordinate males may need to decrease circulating testosterone concentrations in order to avoid harmful fights with dominant males, but increase ejaculate hormone concentrations if it maximizes the success of their rare fertilization opportunities. In such cases, hormone concentrations in the circulation and ejaculates may be the result of a trade-off between the different effects of the hormones. A similar independent regulation has been found for female circulating and maternal T in the yolk of their eggs in birds^9,10^. Unfortunately, data on the correlation between circulating and ejaculate T is surprisingly scarce. In humans, a recent study did not find such correlations for T^11^. In turkeys (*Meleagris gallopavo*), a positive correlation between circulating and ejaculates T concentration was found^12^, whereas a much more recent study did not find a correlation in domestic chickens (*Gallus gallus domesticus*)^7^.

Similarly, not much is known about the possible function of ejaculate T. It is known in mammals that T is needed for sperm cell production by its effect on the testis and increases ejaculate volume by its effect on the seminal vesicles, but to what extent this is related to T concentration in the circulation or ejaculate is actually unknown^13,14^. In human males, a positive correlation was found between T concentration and the number of aberrant sperm cells^14^, but an older study did not find higher T concentration in ejaculates of infertile compared to fertile semen samples^13^, but did find this for 5-alpha-dihydrotestosterone (DHT). Indeed, in mammals T is converted to DHT in the seminal vesicles and blocking this conversion has detrimental effects on the sperm^15^. Testosterone negatively affects metabolism by reducing sperm oxygen uptake, but as this may enhance sperm longevity in the female reproductive tract by reducing the metabolic rate, this effect may actually be positive^16^. Testosterone in the ejaculate may also have an effect on the female as it enters the female reproductive tract such as the sperm storage tubules (SST)^17^ and uterus in chickens, and the fallopian tube and vagina in mice^18–20^. Ejaculate T may also enter the ovum after ovulation, affecting fertilization success or the embryo. In addition, in birds it is known that T deposited in the egg yolk before ovulation, has several short and long term effects on the embryo of that egg^2,8^ and ejaculate T may have an additive effect may add to that effect.

In this study we focused on the effect of ejaculate T in the red junglefowl (*Gallus gallus gallus*) is the wild ancestor of all domestic chickens (*Gallus gallus domesticus*) for the following reasons: like in mammals, this hormone is not only present in the circulation of male chickens, but also in their ejaculates^7^. In the avian female reproductive tract the SST is a specific tissue where sperm is temporarily stored^21^ and which contains sex steroid receptors that play a significant role in sperm storage^17^. If T in the ejaculate reaches the SST it may then induce physiological change in the female and affect her reproductive decision making.

In addition, the red junglefowl is a clear example of T enhancing male conspicuous sexual signals that positively affect female mate choice^22^, as the hormone enhances comb and wattle size, and their colour^23,24^, and increases his attractiveness to females^24,25^. Moreover, in birds, several studies show a positive correlation between male quality as reflected in sexual signals and egg production either in terms of quality or quantity^26–28^. These quality signals also induce differential female yolk androgen deposition (reviewed in^2,8^). This opens the possibility that attractive and non-attractive males not only differ in circulating but also ejaculate T concentration and that the effects found in females are caused by the levels of male hormone affecting the female reproductive tract and not just by its sexual ornaments.

In this study, we firstly report on the difference in plasma and ejaculate T concentration between large combed dominant (attractive) and small combed subordinate (unattractive) males (experiment 1). We assumed that large combed male red junglefowl are usually dominant over small combed males^25,29–31^, and have higher circulating plasma testosterone levels. We firstly checked this assumption in staged dyadic agonistic encounters; fights were set up after which plasma and ejaculate samples were obtained (experiment 1). If social status is related to T production, this may be reflected not only in male circulating T concentration but also in his ejaculate levels. This allowed us to analyse how T concentrations in ejaculates are related to those in the circulation. Secondly, we report on the effects of artificial insemination with T enriched ejaculates on egg production of females (experiment 2). Since yolk T concentration of females is known to affect growth, fear and competitive behaviour of chicks^32,33^, we also investigated whether testosterone enriched ejaculates affected maternal yolk T and androstenedione (A4) deposition, and offspring growth and behaviour.

## Results

### Experiment 1. Social status and testosterone in blood plasma and ejaculates

**Figure 1:**
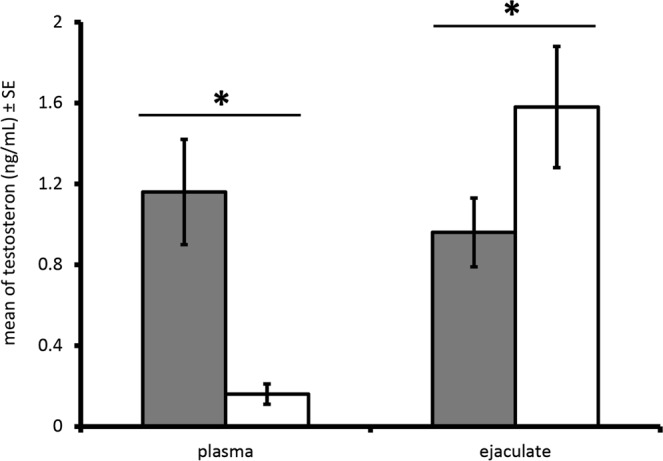
Ejaculate and plasma testosterone concentrations (mean ± SE, in ng/ml) of winners and losers after a fight. Males that won are represented by the dark bars, males that lost by the light bars.

Males that had won a fight had higher plasma T concentrations than males that had lost (winners $\bar{{\rm{X}}}$ ± SE = 1.16 ± 0.26 ng/ml, losers; $\bar{{\rm{X}}}$ ± SE = 0.16 ± 0.05 ng/ml, paired t-test: t = 3.384, df = 4, p = 0.028), but they had lower ejaculate T concentrations (winners $\bar{{\rm{X}}}$ ± SE = 0.96 ± 0.17 ng/ml, losers $\bar{{\rm{X}}}$ ± SE = 1.58 ± 0.30 ng/ml, paired t-test: t = 3.963, df = 4, p = 0.017; Fig. 1). Moreover, losers produced larger ejaculates than winners (winners $\bar{{\rm{X}}}$ ± SE = 575.4 ± 17.7 mg, losers $\bar{{\rm{X}}}$ ± SE = 777.8 ± 36.7 mg, paired t-test: t = 9.062, df = 4, p = 0.0008).

### Experiment 2. Effects of testosterone enriched ejaculates

#### Female egg production

**Figure 2:**
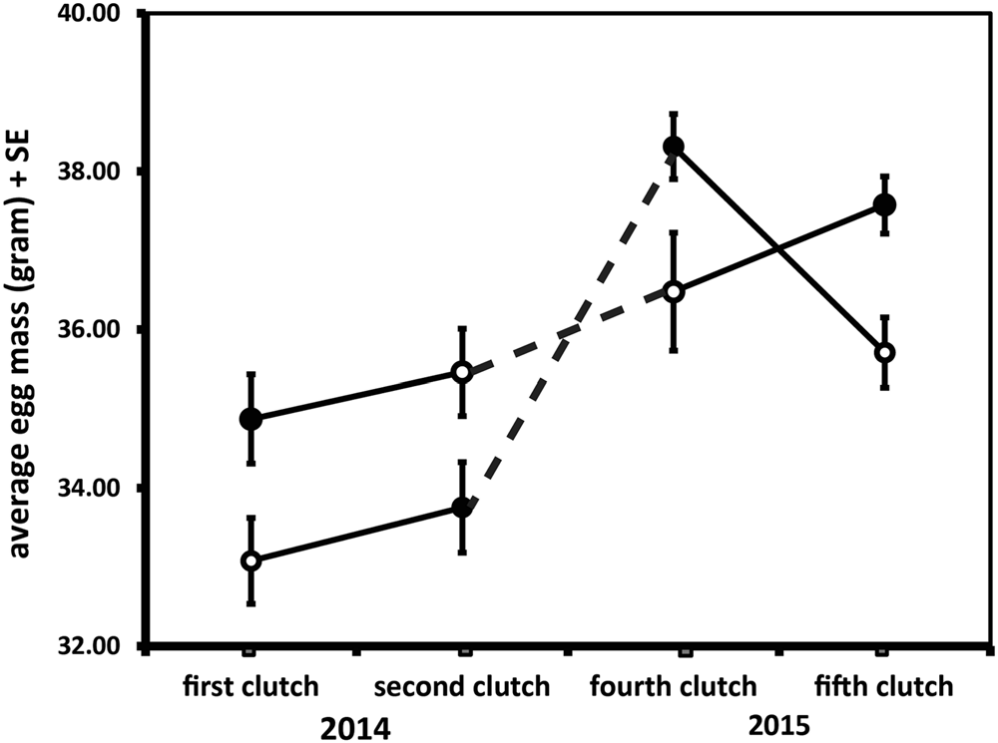
Egg mass (mean ± SE, in gram) for hens first inseminated with testosterone enriched ejaculate (dark symbols) and then with control ejaculate or vice versa (open symbols) in 2014 (n = 26) and with the reverse treatment order in 2015 (N = 24).

There was an effect of testosterone enriched ejaculate (increasing the relative low concentration of ejaculate T of dominant males to the higher level of subordinate males) treatment (TE) and clutch number (order in which clutches were produced) on egg mass, as well as of the interaction between treatment and clutch number (Table 1 row 3, and Fig. 2). In clutch number 1, TE increased egg mass (post-hoc: N_hens_ = 26, N_eggs_ = 141, F _(1,29.99)_ = 7.179, p = 0.012). The effect just reach significance was also present after reversal of the treatment in clutch number 2, but in the opposite direction (post-hoc: N_hens_ = 26, N_eggs_ = 198, F_(1,23.1)_ = 4.354, p = 0.048). Interestingly, the initial positive effect of TE on egg mass was present again in clutch number 4 (post-hoc: N_hens_ = 24, N_eggs_ = 140, F_(1,22.1)_ = 4.449, p = 0.047), and also and very clearly after reversal of the treatment in clutch number 5 (post-hoc: N_hens_ = 24, N_eggs_ = 150, F_(1,21.92)_ = 9.677, p = 0.005). There were no effects of TE or its interaction with clutch number on other reproductive variables (Table 1).

**Table 1:** Female reproductive performance (mean ± SE) per clutch and per treatment with mass variables expressed in grams and hormone values in pg/mg.

	first clutch		second clutch		fourth clutch		fifth clutch		treatment		clutch		Treatment × clutch	
	Treatment	control	control	Treatment	treatment	control	Control	treatment	F	P	F	P	F	P
hens body mass	874.6 (15.8)	872.3 (19.6)	878.4 (16.9)	873.7 (17.5)	855.0 (34.5)	840.8 (27.0)	806.2 (17.9)	789.2 (17.7)	0.004	0.985	2.108	0.105	0.142	0.935
clutch mass	222.6 (21.3)	188.4 (28.0)	300.0 (37.5)	259.0 (38.6)	218.3 (20.8)	220.9 (23.5)	199.3 (11.4)	246.1 (17.4)	0.017	0.898	1.967	0.473	0.290	0.832
egg mass	34.9 (0.56)	33.1 (0.54)	35.4 (0.53)	33.8 (0.60)	38.3 (0.43)	36.5 (0.77)	35.7 (0.43)	37.6 (0.37)	5.970	0.016	17.081	<0.001	5.338	0.002
clutch size	5.9 (0.73)	5.8 (0.89)	8.2 (1.06)	7.5 (1.05)	5.6 (0.51)	6.1 (0.65)	5.3 (0.47)	6.6 (0.49)	0.198	0.657	1.331	0.390	0.871	0.459
yolk mass					13.2 (0.13)	12.7 (0.30)	11.9 (0.25)	12.4 (0.10)	4.644	0.037	12.976	0,008	0.016	0.901
shell mass					4.2 (0.06)	3.9 (0.08)	4.1 (0.05)	4.3 (0.04)	10.168	0.004	1.953	0.169	0.145	0.705
albumin mass					20.9 (0.33)	19.7 (0.50)	19.6 (0.22)	20.8 (0.33)	11.001	0.002	0.070	0.792	0.009	0.921
T concentration					4.9 (0.16)	4.9 (0.21)	5.2 (0.26)	5.1 (0.16)	0.051	0.822	1.448	0.235	0.006	0.937
A4 concentration					75.9 (2.9)	80.8 (9.4)	79.3 (4.5)	75.9 (4.9)	0.477	0.494	0.017	0.898	0.017	0.898

#### Egg composition and fertility

Eggs of TE treated hens had significantly heavier yolk, shell, and albumin mass than eggs from CE hens (Table 1, bottom rows). There were no effects of treatment, clutch number nor the interaction between treatment and clutch number on yolk T or A4 concentration. Treatment did also not affect fecundity. Of the 275 eggs produced in the treatment group 75 showed development after 20 days of artificial incubation, and 36 hatched. Control treated hens produced 229 eggs, of which 77 showed development and 37 hatched. Both the percentage showing development as well as the percentage hatched did not differ between the groups (proportion test, egg development: Z = 1.453, p = 0.147; hatching: Z = 0.852, p = 0.397).

#### Chick growth, competitiveness, and stress sensitivity

**Figure 3:**
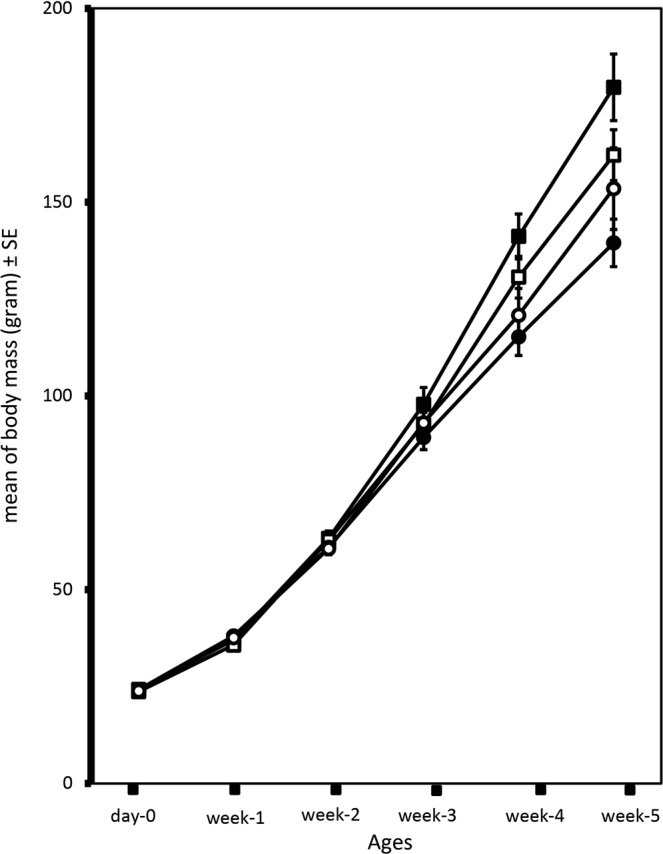
Body mass (mean ± SE, in gram). The sons of TE (dark square) were heavier than CE (light square) and daughters of TE (dark circle) were lighter than CE(light circle).

**Figure 4:**
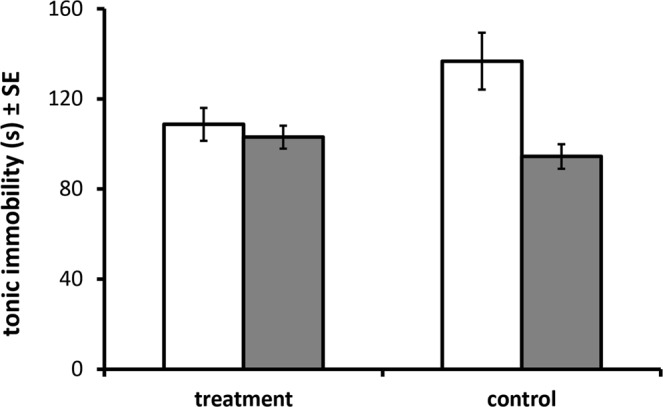
Tonic immobility (mean ± SE, in second). The mean of TI of TE chicks and CE chicks for sons (light bars) and daughters (dark bars).

There was a significant interaction of offspring sex with treatment and age on growth over the first five weeks of life (N = 59, F _treatment_ × _sex_ × _age(5,275)_ = 2.880, p = 0.015, Fig. 3), which resulted in larger body mass in sons at weeks 5 (N = 59, F_treatment_ × _sex,(1,55)_ = 4.088, p = 0.048) and lower body mass in daughters of TE females compared to CE females. There was no effect of treatment on either the number of mealworms eaten (N = 64, F_same-sex(1,60)_ = 0.128, p = 0.772; N = 49, F_opposite-sex (1,45)_ = 1.154, p = 0.288, Fig. 1 in SI section 8) or the number of individuals that ate the most mealworms in same-sex or opposite-sex competition tests: 53% of the TE chicks ate more than the CE chicks in same-sex trials (N = 64, Z_same-sex(1,60)_ = −1.142, p = 0.253), and 41% of the CE chicks ate more in the opposite-sex trials (N = 49, Z_opposite-sex(1,45)_ = 0.383, p = 0.708). There was however a significant interaction of offspring sex and treatment on the duration of tonic immobility (N = 59, F_treatment_ × _sex,(1,291)_ = 11.789, p = 0.001, Fig. 4). Treatment decrease significantly the duration of tonic immobility in sons (N = 28, F_(1,138)_ = 10.551, p = 0.001), but did not effect in daughters (N = 31, F_(1,153)_ = 1.757, p = 0.187).

## Discussion

Male social status was assessed by dyadic fights between males that differed in comb size, a secondary sexual ornament known to be dependent on circulating plasma testosterone^23^. As predicted^23,34^, males that had on average a larger (also relative) comb size, but were also overall on average significantly heavier, won all fights and became dominant over males with smaller comb sizes. These large combed males had, in line with other studies^31^, elevated circulating T concentration compared to their smaller combed rivals. On the other hand, ejaculate T concentrations were lower in large combed winners compared to small combed losers. This indicates that ejaculate T concentration is not merely a reflection of T concentration in the circulation, suggesting a trade-off in the male between allocating this hormone to their own body and to their ejaculate. Dominant red junglefowl males can monopolize copulations by means of T dependent behaviour and sexual ornaments, whereas subordinate males may benefit from signalling submission to avoid costly fights and only occasionally ‘steal’ copulations from dominant males. Subordinate males may benefit from lower circulating T concentration due to e.g., reduced social stress, and higher ejaculate T by enhancing the chance their copulations result in fertilizations. However, whether T in the ejaculate increases fertilization is as yet unclear. On the other hand, indirect evidence supports this. Several studies indicate that dominant roosters (with significantly larger combs, and as we have shown, lower T concentration in the ejaculates) produce sperm with lower motility than subordinate roosters^35–37^. In addition, males with smaller combs (having in our study elevated ejaculate T concentration) had the highest percentage of viable sperm^38^. Also, another study showed that sperm quality (mobility in this case) of males that became subordinate after a social challenge was higher than that of males that became dominant^39^. Higher sperm mobility and viability increase the possibility to win when in competition to fertilize eggs^36,40,41^. However, we did not test the competitive advantage of TE over CE. This would be an interesting topic for a follow up study that would entail a much more elaborate experimental set up including molecular paternity analysis.

On the other hand, the literature on the direct relationship between ejaculate T concentration and sperm quality is inconsistent. In humans, seminal T concentration can be both positively or negatively correlated with sperm quality^14,42^. In nellore bulls (*Bos indicus*) there was a negative correlation of seminal T concentration with the number of sperm deficits^43^, but another study found no such correlations at all^44^. In birds, the literature is very scarce: In domestic chickens (*Gallus gallus domesticus*), testosterone may induce negative effects on sperm metabolic rate. This may increase their longevity due to lower oxidative stress but may also decrease swimming speed due to the reduced ability of sperm cells to take up oxygen^16^. In turkeys, there was no relationships between seminal T concentration and ejaculate variables (concentration and total number of spermatozoa)^12^. In our study, it did not seem to affect female fecundity as the percentage of eggs with development and the number of chicks hatched did not differ between treatments.

Another way to enhance fertilization success is to increase ejaculate volume and sperm cell number. Testosterone is indeed known to affect both, through its effect on the seminal vesicles in mammals and the seminal sac in birds^45,46^. When we manipulated males via abdominal massage in order to collect their ejaculates, we received about 1.4 times more ejaculate from losers than from winners. Moreover, male turkeys that produced high ejaculate volumes (about 2.4×) increased the number of sperm by 3 times compared to low volume producing males^12^. This suggests that less attractive males produce larger ejaculates with a higher sperm count than attractive males in order to increase the chances to sire offspring in the few mating opportunities they may have. Finally, elevated ejaculate T concentration may also induce females to more efficiently store sperm by acting on the sex steroid receptors of the sperm storage tubule^17^. The receptors known today respond not only on progesterone and oestrogen but also on testosterone^47–49^. How this independent regulation of T in the male’s ejaculate and circulation comes about is as yet unclear. Potentially, differential transport proteins, blood vessel constriction or dilatation, or hormone metabolism may play a role. It is interesting that in mammals the seminal vesicles contain receptors for luteinizing hormone^15^, which suggests that these vesicles may produce T themselves explaining the lack of correlation between circulating and ejaculate T concentration.

When receiving T enriched ejaculates females produced overall heavier eggs, with heavier yolks, eggshell, and albumin mass than when receiving control ejaculates. As enriched ejaculates mimic the ejaculates of subordinate males, our result seems in line with a study in zebra finches (*Taeniopygia guttata*) in which it was shown that females laid larger eggs when paired with sexually unattractive males, which may perhaps function as compensation for low genetic mate quality^50^.

Unexpectedly the reversal of treatment in the first year of our study did not result in a reversal of the effect on egg mass, but it did so in the second year. This lack of effect in the second clutch of the first year was unlikely to be an effect of a difference in body mass, since the groups were matched for mass. It may, however, have been caused by a carry-over effect of the T enriched ejaculates in the first clutch since we switched the treatment immediately after clutch completion. Females in that first clutch receiving TE and then CE would then still produce relatively large eggs in the second clutch as an after effect, whereas females that first received CE and then TE may have simply reacted as the others that had received TE first. To avoid a possible carry-over effect of treatment in the repeat of the experiment in the spring of 2015, we left the females to recuperate for one week and the hens in the CE to TE group increased egg mass, while the females of the TE to CE groups decreased the mass of their eggs. Apart from this, in the second year of the experiment egg mass increased for all birds. This was expected since young females often produce smaller and lighter eggs than older females (e.g.^51^).

How T in the ejaculate affects egg mass is as yet unknown. In fruit flies and birds, the oviduct and storage tubule are innervated by the central nervous system^52,53^. In birds, T binding to the steroid receptors in the oviduct may be transduced to signals to the brain, affecting egg production. Although eggs contain many chemical compounds, we concentrated on yolk androgens since it is known that females may differentially deposit these hormones in their yolks based on mate quality (reviewed in^8^). Moreover these hormones are well known to affect offspring growth and behavioural development^2,8^. Yolk T, may be higher after insemination with elevated levels of T because, being a lipophilic molecule, ejaculate T may be entering the fatty yolk in the female tract during fertilization before the addition of albumin and shell.

Testosterone enriched ejaculates affected offspring growth during the first five weeks after hatching, but did so in a sex-specific way. The sons of TE were heavier than CE, and the daughters of TE were lighter than CE. This sex-specific effect of TE also appears in tonic immobility. The TI of TE sons was shorter than CE sons, but this was not the case in daughters. The longer duration of tonic immobility indicates a higher level of corticosterone which may be related to chronic stress and the retardation of the muscle growth^54^. As TE males grew faster than control males, but the lower body mass in TE versus control females was not significant, this might explain the TI data. However, the sex-specific effect of testosterone enriched ejaculate on growth and TI may have been facilitated by egg mass, although due to the small sample size we could not detect effects of treatment or differences between the sexes. The effects cannot be explained by differential androgen deposition in the eggs, since there were no differences in either yolk T concentration or yolk A4 concentrations between the treatments (which may explain the lack of behavioural differences between TE and CE chicks). Unless the treatment induced sex specific hormone deposition in the eggs, for which currently there is some evidence^8,55,56^, but see^57^. This would require that females know the sex of their eggs before laying. In that case another possibility is that the mothers prenatally affect growth of sons and daughters differently (e.g. by differential deposition of nutrients to the egg) according to the hormonal signal she received in the ejaculate. Alternatively, male and female embryos utilize hormones with a different rate in the early stage of embryogenesis. Another possibility is that young chicks may be differentially sensitive to maternal or paternal signals.

In conclusion, we demonstrated for the first time that social status has an effect on both plasma and ejaculate T concentrations, showing an inverse relationship to each other. This relationship suggests a possible trade-off where subordinate males decrease their circulating T to avoid attacks from dominant males and increased their ejaculate T to compensate for poor male quality. The increase of ejaculate T concentration to enhance offspring growth indicates a more complex and adaptive regulatory mechanism than often assumed. Testosterone enriched ejaculates also had an effect on the female’s reproduction, and offspring growth and behaviour. These new results indicate an intriguing interaction between a cryptic paternal trait and maternal effect resulting in the offspring sex-specific growth and behaviour. Our results open a new avenue for investigating mechanisms underlying male hormone allocation, female reproductive decisions, and the effects on offspring traits as well as the fitness consequences of ejaculate T concentration and its relevance for intra and inter-sexual conflict over reproduction.

## Materials and Methods

### Animal experiment

We used captive bred red junglefowl from our facility at the University of Groningen, The Netherlands, which is an outbred population of wild caught birds coming originally from Asia. All males displayed the species-specific calls, much shorter than those from domestic birds, and most birds still moulted their neck feathers in autumn. We performed a power analysis based on data of an earlier experiment where we found an effect of dominance on egg mass. This revealed (power 0.8, alpha 0.05) the sample size we used for the number of pairs. Since we needed chicks of both sexes from each pair, we aimed for at least 3–4 eggs from each pair that would successfully hatch, taking into account the level of hatching success in earlier experiments using this species. The welfare of all birds was assessed on a daily basis throughout the experiment. The animal observations were done for 30 minutes during the male-male confrontations and before and thereafter all birds were checked two times a day. All handling and treatment of animals were carried out by experienced scientists and animal caretakers with a licence to perform animal experiments.

All experimental procedures were carried out according to the regulation of Dutch law for laboratory animals and approved by the animal experimentation committee of the University of Groningen the Netherlands (licence DEC 6710 C in 2014).

### Experiment 1: Social status and testosterone in blood plasma and ejaculates

#### Experimental design

To determine T concentration of males of different social status, we selected 10 males (±2.5 years old) on the basis of comb size and body mass such that 5 potentially dominant males had a larger comb size and a larger body size than 5 subordinate chickens (see SI Section 1). Before the experiment, all 10 males were housed in the multi-male group. The predicted winners (large combed and heavy males) and predicted loser (small comb and light males) were allocated to one of 10 identical aviaries ((1.5 by 4 by 2 m) (w × l × h)) four days before the experiment, containing ad lib food, grit and water. After 4 days, randomly chosen small combed males were introduced into the cages of the predicted winners. In the first 30 minutes after introduction each pair was observed to determine the dominance relationship between the males. Dominance was quickly established within 15 minutes and often within 5 minutes. Dominance was determined by observing aggressive approaches (raised neck feathers), lateral display, crowing, and overt aggression, and by withdrawal and freezing as a reaction of the subordinate males. Twenty-four hours later an ejaculate sample and a blood sample were obtained in which T concentration were determined (see SI section 2, 3, and 6). Ejaculate samples were weighed before hormone analyses. As expected, all the preselected winners were dominant over the preselected losers. Male biometry is outlined in Tables A and B of the SI section.

### Experiment 2: Effects of testosterone enriched ejaculate

#### Experimental design

Since winner males had lower T concentration in their ejaculates than loser males, we investigated whether ejaculate T concentration affected female reproductive investment. Prior to the experiment, the chickens were housed in all female or all male flocks. Male and females were about one year old (8–10 month) and sexually naïve at the start of this experiment. Hens were assigned to a treatment condition such that there were no differences in body mass between the control and treatment groups (see Table 1). Thirteen potentially dominant and subordinate males were selected in 2014 and twelve pairs in 2015 from our stock and housed in identical aviaries (see above). The criteria for potential dominant males was based on their body mass and comb size as above. Male biometry was determined before the first and the second part of experiment 2. Each male was housed in a triplet cage, i.e. the male was placed in the middle of three identical aviaries, between two females that were physically but not visually separated from the male by wire mesh. In order to strengthen the perceived attractiveness of the males in such a triplet, each male was confronted with an intruder male for 15 minutes (one day after the initial housing event) and left to exert its dominance over the intruder in full view of the females. This procedure was repeated every week during the entire experiment. This procedure was carefully monitored for (1) animal welfare reasons and (2) to check whether the resident male indeed was dominant over the intruder, which was always the case. The males in the middle of the triplet were the ejaculate donors for the insemination procedure.

Each female in a triplet was either inseminated with a T enriched ejaculate (TE) or received the control treatment (CE) every other day (for treatment details on T concentration see below). Females were allowed to lay a clutch (clutch number 1, autumn 2014) and we recorded female reproductive investment (clutch size, egg mass, and clutch mass). Eggs were collected every afternoon and individually marked with non-toxic ink and stored at room temperature (see SI section 5). Directly after clutch completion, the treatment was reversed and the same variables were recorded (clutch number 2). After the second clutch, the treatment was reversed again. Females were allowed to produce a third clutch (number 3). Eggs from this clutch were collected and artificially incubated in order to assess whether there were any effects of the treatment on offspring growth and behaviour.

Newly hatched chicks from clutch number 3 were housed in cages (100 cm × 100 cm × 100 cm) in our inside facility under LD 14:10 h, 60% relative humidity, and a room temperature of 24 °C. Chicks were randomly housed in groups of 5 to 10 (average 8) according to laying date and mixed across treatments (and sex). Each cage was fitted with a red heat light. Food and water were provided in ad libitum quantities. Individual birds were fitted with a rubber colour band directly after hatching and at three weeks old they received a numbered metal wing clip through the patagium. At this age, the sex of the chicks could be determined based on the size of the comb and the feather colour in the neck and chest region.

**Figure 5:**
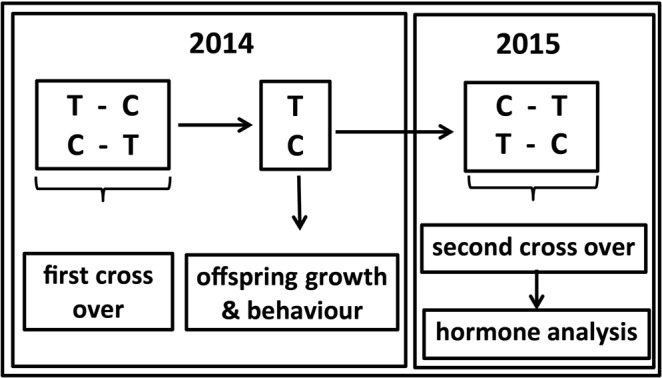
Schematic representation of experiment 2. In total hens produced 5 clutches in either the TCTCT condition or CTCTC condition where T stands for insemination with testosterone enriched ejaculate and C for insemination with control ejaculate. Time runs from left to right. The third clutch is not included because it was used to determine offspring growth and behaviour.

The experiment was repeated the following spring (2015). The hens of the TE treatment in the previous group were switched to the CE treatment and vice versa (see Fig. 5). All females remained matched to the same males. Females treated with TE and CE were allowed to produce a clutch again (clutch number 4). After clutch completion females were left to recuperate for one week after which treatment was reversed once more and the hens were left to produce a fifth clutch. The eggs of clutch 4 and 5 were collected for the analyses of yolk T and androstenedione (A4) deposition (see SI section 3 and 6). This repetition involved 24 of the original 26 hens from the experiment in 2014 (2 hens had died during the winter). The overall design is schematically illustrated in Fig. 5.

#### Ejaculate treatment protocol

After housing males and females in triplets, we collected fresh ejaculates from all the dominant males every other day. Ejaculates were pooled in a vial (±4 ml), and then half of the ejaculate was enriched with 0.5 ng testosterone suspended in 30 µl sesame oil per 0.17 ml ejaculate (TE). This increased the relative low testosterone concentration of the ejaculate of winners (see experiment 1) from 0.96 to 3.31 ng/ml (total amount was 0.66 ng hormone, as 0.2 ml was used for insemination represent the ejaculate volume of normal copulation) which is within the physiological range of the testosterone concentration in the ejaculate of losers (close to the average plus 2 times the standard deviation of the mean). The other half of the females received 0.17 ml ejaculate mixed with 30 µl sesame oil only (CE). Because of the addition of the sesame oil, control birds received a slightly lower T concentration, from 0.96 to 0.82 ng/ml, than that of dominant male ejaculates (total amount per insemination: 0.16 ng). To maintain the ejaculates at body temperature (±42 °C for red junglefowl), vials were kept in a water bath with the same temperature until artificial insemination (directly after mixing), which was between 60–120 minutes after ejaculate collection.

For artificial insemination, hens were caught and held in an inverted position in which the muscle around the ischium was gently massaged. Once the upper and lower lip of the cloaca opened, a syringe was inserted, and females received 0.2 ml of either T enriched ejaculate mix or the control mix. Each female was inseminated every other day for three weeks or until clutch completion. Inseminations took no longer than 3 minutes and were habitually performed between 10.00, and 12.00 am after most hens had laid an egg.

#### Behavioural tests

We tested the chicks in two behavioural paradigms: (1) a food competition test (age 4–8 days) and (2) a tonic immobility test (age 21 days), see also SI section 4. In the food competition test (n = 59) we tested both same-sex and opposite-sex dyads with 30 chicks from T enriched ejaculate treated mothers and 29 chicks from control mothers. We used the 59 survivors for the TI test and for the analysis of growth until five weeks of age. For the competition test we marked control chicks such that they could be recognized during the video analyses. In each competitive test, a pair of focal birds (two individuals from different treatments) were placed in a circular arena (diameter = 80 cm) behind a wire barrier. Then a mealworm (*Tenebrio molitor*), a very attractive food item for the chicks, was placed on the other side of the barrier. As soon as both chicks showed interest, the barrier was removed and they were left to compete for the item which occurred in all tests. The test ended when the meal worm was eaten. For each pair 5 such trails were conducted. The total number of mealworms obtained by each individual was summed over the five trials. We also determined which chick ate most of the mealworms over the five trials.

At the age of three weeks we performed a tonic immobility test^58,59^. The tonic immobility test was performed blindly with respect to sex and treatment. The tests were performed in the same room where the chicks were housed in, to avoid additive effects of a novel environment. Chicks were placed on a table in the middle of the room and held on their back for approximately 10 seconds, then the researcher slowly retracted his hand and the time it took for the chick to be fully upright again was recorded. All animals were tested in five consecutive trials.

Body mass was determined directly after hatching and then every week until they were 5 weeks old. After five weeks, all chicks were moved to a single large aviary.

#### Statistical analyses

The data of differences between winners and losers in plasma and ejaculate T concentration, body mass, comb size and the comb size relative to body mass were tested with paired t-tests.

The effect of treatment on egg mass (data of 2014 and 2015 were pooled) was tested using linear mixed models, with each egg nested in clutch and clutch nested in mother as a random effect. Models included clutch number (order in which the clutches were produced), treatment and the interaction between clutch number and treatment as fixed effects. Post-hoc tests were fitted for differences within clutches using linear mixed models with each egg nested in mother as a random effect.

For effects of treatment on other reproductive variables (clutch size, clutch mass and hormone concentration), the data were analysed using linear mixed models with clutch nested in hen as a random effect. For the egg components: albumin, shell, yolk mass and hormone concentration, the analysis was only conducted on data from 2015 (no data was collected in 2014), using clutch number 4 and 5. The linear mixed models included clutch, treatment and the interaction between clutch and treatment as fixed effects.

In the offspring growth analysis, the dependent factor was the body mass of chicks (gram) at six different time points from the day of hatching until 5 weeks of age. The data were analysed using linear mixed models with treatment, sex, age and the interaction between treatment, sex and age, as fixed effects and chick nested within mother as a random effect. A post-hoc test for body mass at 5 weeks after hatching was fitted using a linear mixed model with treatment, sex, and the interaction between treatment and sex as fixed effects and chick nested within mother as a random effect.

The effect of treatment on tonic immobility was tested using a linear mixed model, with each trial (5 times) nested in chick as a random effect. Models included treatment, sex and the interaction between treatment and sex as fixed effects. A post-hoc test for the duration of tonic immobility based on the sex was fitted using a linear mixed model, with each trial nested in chicks as a random effect and treatment as fixed effect. The residuals of the mixed models were checked for normality (see Supplementary Data Set). The effect of treatment on the performance in the competition test between the chicks of the two treatments was analysed as follows: we first fitted a univariate analysis of variance on the difference within each chick pair, in the number of mealworms eaten, separately for same-sex and opposite-sex trials with the difference in body mass (TE-CE) as a covariate, and calculated whether the intercept differed from 0. Subsequently we tested in same-sex and opposite-sex competitive trials whether the number of birds that ate the majority of the mealworms offered, differed between the treatments using proportion tests.

All statistical analyses were performed with SPSS23. and the models have been tested for normality using their residual values (see Supplementary Data Set).

## Supplementary information

Suplementary information

## Data Availability

Our data will be deposited at Dryad after the manuscript has been accepted.
