# Supplementary material for: Ejaculate testosterone levels affect maternal investment in red junglefowl (Gallus gallus gallus)
Source: Sci Rep. 2019 Aug 20;9:12126. doi: 10.1038/s41598-019-48563-w (PMC6702165; doi:10.1038/s41598-019-48563-w)
Supplement: Supplementary file 1 — Suplementary information [file 41598_2019_48563_MOESM1_ESM.pdf]

Ejaculate testosterone levels affect maternal investment in Red junglefowl (*Gallus gallus gallus*).

Asmoro Lelono[1*,2], Bernd Riedstra[1], Ton Groothuis[1]

[1] Behavioural Biology, Groningen Institute for Evolutionary Life Sciences, University of Groningen, Nijenborgh 7, 9747 AG, Groningen, The Netherlands.
[2] Department of Biology, Faculty of Mathematics and Natural Sciences, University of Jember, 68121 Jember, East Java, The Republic of Indonesia. 9
[*] Correspondence and requests for materials should be addressed to A.L (e-mail: a.lelono@rug.nl).

Supplementary Information

Method

1. Biometry

Biometrical measurements of all males were taken just before housing them in the experimental condition of both experiment 1 and 2. Body mass was measured to the nearest gram. Rooster combs were photographed using a digital camera (Canon SX 500 IS: diaphragm 4.3 – 129.0 mm). For reference purposes, a round sticker (d = 0.5 cm) was attached to the comb before photographing. Pictures were taken of only the left side of the rooster heads. The surface area of the comb (comb size) was determined by contrasting the number of pixels in the comb against the number of pixels in the sticker from the same photograph. The number of pixels was determined by manually outlining the comb and sticker using GIMP 4.8. Comb length was determined by measuring the imaginary straight line extended from the point of separation at the base of a chicken's beak and comb above the nostrils to the farthest point of the outer parts of the comb. For experiment 2, all hens were weighed at the beginning of the experiment and treatment groups were matched for body mass. We calculated comb size relative to body mass by dividing comb size by body mass and tested the difference between dominant and subdominant birds of the original 5 pairs used in experiment 1 and 13 in experiment 2 by a paired t-test. This was highly significant ($T_{(1,17)} = 2.07$, $p = 0.001$, for details see table A and B).

2. Ejaculate collection and blood sampling

Ejaculate was collected as follows: All roosters were habituated to the procedure to produce an ejaculate to a handler (A.L.) using the abdominal massage method [29]. In short, the cloaca of a rooster was massaged with the thumb on one side, and the

fingers on the other side, while the back and tails were gently stroked with the other hand. Usually, after about half a minute an ejaculate was released and caught in a tube. If the ejaculate was not released, the cloaca was massaged for at least two minutes more.

We collected about 1 ml blood from the *vena ulnaris* within 3 minutes after opening the door of the aviary using a syringe with a 25G needle. The tube in which the blood samples were collected, were pre-rinsed with EDTA (9 g/100ml) to prevent coagulation. Samples were then centrifuged for 10 minutes at 2000 g, and circa 0.5 ml of plasma was then separated and stored at -20°C until analyses. The ejaculate and plasma samples were weighed before hormone analysis.

3. Hormone assays (ejaculate and plasma)

Plasma and ejaculates concentrations of T were quantified by radioimmunoassay (RIA). To extract the hormones, 500 mg of plasma and 395 mg of ejaculate were weighed (accuracy 1 mg), 300 µl of milliQ water was added and 50 µl of 3H-labeled testosterone (NET553, Perkin Elmer) was added to trace the recovery of extracted hormones during the extraction procedure. This solution was incubated for 15 minutes at 37°C before being extracted in 2 ml of diethyl ether/petroleum ether (DEE/PE, 70/30 v/v) by vortexing for 60 seconds. Extracted samples were centrifuged at 2000 rpm for 3 minutes (4°C) to separate the ether phase, the samples were snap-frozen and the ether/hormone phase decanted into a fresh tube. The extraction procedure was repeated twice with an additional 2 ml of DEE/PE, vortexed for 30 seconds and 15 seconds, respectively. Next, the extracts were dried under nitrogen at 37°C. Hormone extracts were rinsed in 2 ml of 70% methanol to precipitate any lipids and stored at least overnight at -20°C. Subsequently, the tubes

were centrifuged, decanted into a fresh tube, re-dried under nitrogen at 50°C and stored at -20°C.

Before assay, extracts were thawed and dissolved for plasma in 250 µl phosphate-buffered-saline with gelatin, and 100 µl PBSG for the ejaculate (PBSG: 5.3 g NaH_2PO4. H_2O; 16.35 g Na_2HPO4. $7H_2O$; 9.00 g NaCl; 1.00 g gelatin; 1.00 g $NaN_{3)}$. Recoveries of the initially added labelled T were measured in a subsample of this solution using scintillation cocktail (Ultima Gold, Perkin Elmer) and radioactivity counted on a liquid scintillation counter. The average recovery was 78% for plasma (SD 1.8 %) and 77% for ejaculate (SD 2.9 %) Subsequently, 25 µl of the extracted sample was used for T determination using a commercial RIA kit (TESTO-CT2, Cisbio Bioassays, Codolet, France). The antibody used had a cross reactivity of 2.6% for 5α-dihydrotestosterone. Standards were prepared using dilution series from pre-prepared stock and ranged from 0.08-20 ng/ml T.

Experiment 2: Effects of testosterone enriched ejaculate (TE)

4. Behavioural tests

We tested the chicks in two behavioural paradigms: 1) a food competition test (age 4 – 8 days) and 2) a tonic immobility test (age 21 days). At the time we performed the food competition tests, It was not possible to differentiate between male and female chicks. We tested each pair of TE-CE chicks randomly and individual chicks were used several times over several days to increase the chance of having ample same-sex and opposite-sex combinations. Each competition test consisted of five consecutive trials. At three weeks of age when sex could reliably be established, we retrospectively distinguished between same-sex and opposite-sex competition tests since the chicks were individually marked. In total, 59 of 73 chicks that hatched were

used for both behavioural test. Ten chicks could not be properly matched for age as not all chicks hatched synchronously and 4 died during the course of the experiment (before the sex of the chicks has been determine). These 59 chicks consisted of 30 chicks from T enriched ejaculate treated mothers and 29 chicks of the control mothers. We used the data of these 59 chicks for TI and growth analysis. In the competitive test we testes each pair in 5 consecutive trials. Each chick was tested 4 times (each day once for 4 successive days) each time against different opponent. When the sex could reliably be determined we had recorded 64 combination of same-sex tests (33 female vs female and 31 male vs male) and 49 combination of opposite-sex tests (31 female vs male and 18 male vs female).

5. Female egg production

To determine whether variation in egg characteristics was influenced by the treatment, we started egg collection seven days after the first insemination, so that all eggs were exposed to the treatment during the entire phase of rapid yolking, which typically lasts 6 days. We recorded egg production on a daily basis. Eggs were weighed and individually marked, and removed from the aviaries every day.

6. Egg yolk hormone analyses

After collection and measuring, all the eggs of clutch number 4 and 5 were stored at -20°C in order to determine yolk T and A4 concentration. Yolks were manually separated from the shell and albumen, and their individual weights were recorded. Hormone concentrations were quantified by radioimmunoassay (RIA). To extract the hormones, 200 mg of yolk/milliQ water mixture (1+1) was weighed (accuracy 1 mg), 300 µl of milliQ water was added and 50 µl of ^{3}H-labeled testosterone (NET553,

Perkin Elmer) was added to trace the recovery of extracted hormones during the extraction procedure. This solution was incubated for 15 minutes at 37°C before being extracted in 2 ml of diethyl ether/petroleum ether (DEE/PE, 70/30 v/v) by vortexing for 60 seconds. Extracted samples were centrifuged at 2000 rpm for 3 minutes (4°C) to separate the ether phase, the samples were snap-frozen and the ether/hormone phase decanted into a fresh tube. The extraction procedure was repeated twice with an additional 2 ml of DEE/PE, vortexed for 30 seconds and 15 seconds, respectively. Next, the extracts were dried under nitrogen at 37°C. Hormone extracts were rinsed in 2 ml of 70% methanol to precipitate any lipids and stored at least overnight at -20°C. Subsequently, the tubes were centrifuged, decanted into a fresh tube, re-dried under nitrogen at 50°C and stored at -20°C. Prior to assay, extracts were thawed and dissolved in 450 µl phosphate-buffered-saline with gelatine (PBSG). Recoveries of the initially added labelled T were measured in a subsample of this solution using scintillation cocktail (Ultima Gold, Perkin Elmer) and radioactivity counted on a liquid scintillation counter. Average recovery was 88% (SD 2.4 %). Subsequently, 25 µl of the extracted sample was used for T determination using a commercial RIA kit (TESTO-CT2, Cisbio Bioassays, Codolet, France). The antibody used had a cross reactivity of 2.6% for 5α-dihydrotestosterone and 1.7% for androstenedione. Standards were prepared using dilution series from pre-prepared stock and ranged from 0.08-20 ng/ml T.

For determining A4 concentration we used 50 µl of extracted sample (dilution x16) and a commercial kit (DSL-3800, Beckman Coulter GmbH, Sinsheim, Germany). Standards were prepared using dilution series from pre-prepared stock and ranged from 0.16-20 ng/ml for A4. ‘Pools’ of yolks were used as external controls and intra assay variation was 1.98 % for T and 4.1% for A4.

7. Table

Table A. Experiment 1: Biometry of roosters that either won (N=5) or lost (N=5) staged pairwise agonistic encounters. Variables are expressed as means (± SE), the t- and p-values refer to the outcome of paired t-tests on the variables in the first column.

	losers	winners	t	p
body mass (g)	1029.4 (36.1)	1165.4 (47.7)	2.27	0.053
comb size (cm^2)	9.97 (0.26)	15.02 (1.23)	2.84	0.022
comb length (mm)	59.66 (1.31)	66.96 (2.21)	3.99	0.004
comb size/body mass	0.010 (0.002)	0.013 (0.001)	-1.21	0.292

Table B. Experiment 2: Mean (± SE) of body mass and comb characteristics of dominant (winners) and subordinate (losers) roosters (N=13 pairs in 2014, N= 12 pairs in 2015), the t- and p-values refer to the outcome of paired t-tests on the variables in the first column.

		losers	winners	t	p
year 2014	body mass (g)	997.3 (25.1)	1227.4 (17.4)	7.513	<0.001
	comb size (cm^2)	7.6 (0.70)	15.7 (0.56)	8.959	<0.001
	comb length (cm)	61.3 (1.79)	71.6 (1.33)	4.634	<0.001
	comb size/body mass	0.007 (0.001)	0.013 (0.002)	-5.82	<0.001
year 2015	body mass (g)	1100.0 (36.4)	1351.8 (26.3)	5.598	<0.001
	comb size (cm^2)	15.0 (0.64)	18.4 (0.47)	4.244	<0.001
	comb length (cm)	62.3 (1.09)	75.1 (1.91)	5.773	<0.001
	comb size/body mass	0.014 (0.002)	0.014 (0.004)	-0.076	0.094

8. **Figure**

Figure 1.The difference in the number of mealworms eaten between TE and CE chicks in same-sex and opposite sex food competition tests.

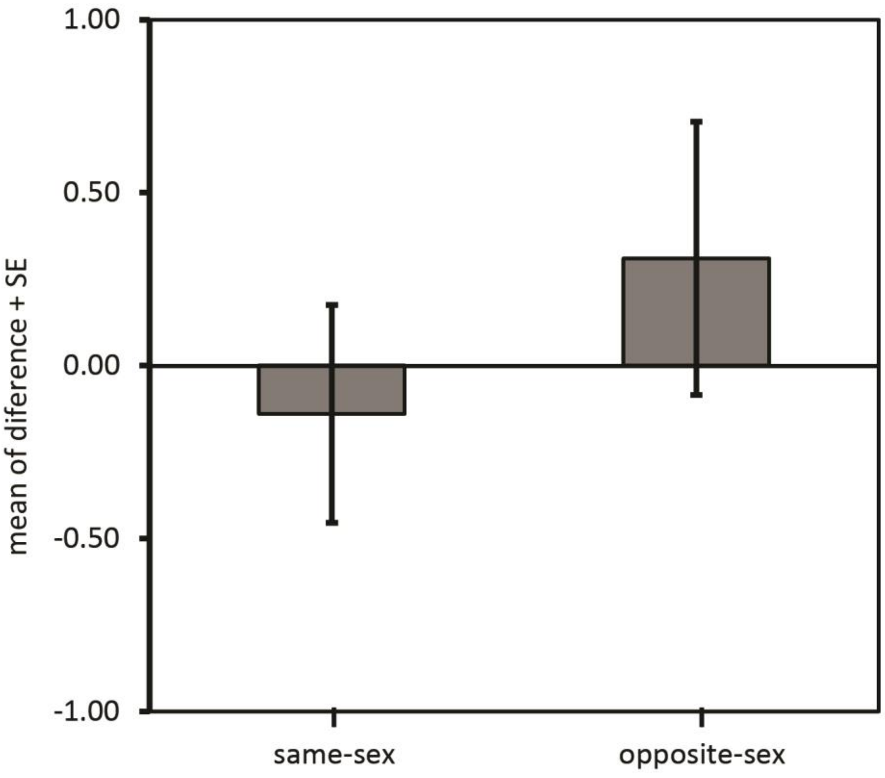
1.00
0.50
0.00
-0.50
-1.00
mean of diference + SE
same-sex
opposite-sex
